# High-throughput sequencing data of the microbiota and antibiotic resistance genes from biofilms on polystyrene and nylon rope incubated in Bergen harbor

**DOI:** 10.1016/j.dib.2025.111718

**Published:** 2025-05-28

**Authors:** Didrik H. Grevskott, Manish P. Victor, Tyra E. Lima, Vera Radisic, Priyank S. Nimje, Nachiket P. Marathe

**Affiliations:** a Department of Contaminants and Biohazards, Institute of Marine Research (IMR), Bergen, Norway; b Department of Biological Sciences, University of Bergen, Bergen, Norway

**Keywords:** Biofilms, Metagenomics, Bacterial diversity, Antibiotic resistance genes, Plastic polymers, Microplastics

## Abstract

Plastics can provide a hydrophobic surface for microorganisms to attach, colonize and form microbial biofilms, referred to as ‘plastisphere.’ The aim of this study was to determine the microbiota of biofilms on plastic, using field trials in Bergen harbour, Norway using metagenomics. Polystyrene (PS) and nylon ropes (PA) were submerged in sea for four weeks, biofilm communities were collected, and the extracted DNA was subjected to metagenomic sequencing (n=12). The average salinity and temperature during the experiment were 9.02 °C (8.2-10.7) and 28.85 **‰** (26.1 **‰** - 33.1 **‰**). We obtained a total of ∼460 Gigabases of sequence data from our samples. Gammaproteobacteria and Alpha proteobacteria were the most prominent on these polymers with β-lactamases as the most abundant resistance gene class. The datasets will be useful for the scientific community working on plastic-associated biofilms.

Specifications Table

**Table:** 

Subject	Biology
Specific subject area	Microbiology and genomics
Type of data	Figure and Shotgun metagenomic sequencing data
How data were acquired	Biofilm samples were collected from the polystyrene and plastic rope surfaces submerged in theSea, at Bergen, Norway (60°40’04.35” N 5°30’63.33” E)
Description of data collection	Genomic DNA was extracted from the biofilm samples using the DNeasy Power Water kit (Qiagen, Germany) following the manufacturer’s protocol. The extracted DNA was quantified, using dsDNA High Sensitivity (HS) assay kit on the Qubit® Fluorometer (Thermo Fisher Scientific, USA). Shotgun metagenomic library were prepared, using Nextera Library Prep kit (Illumina, USA). Sequencing was performed, using Illumina NovaSeq 6000 platform (Illumina, USA), 150 bp paired end reads, at the Norwegian Sequencing Centre (Ullevål University Hospital, Oslo, Norway).
Data source location	The raw sequencing data have been deposited in the Sequence Read Archive (SRA) under the BioProject accession no. PRJNA1114926 and publicly available under the following accession numbers (SRR29182337-48).

## 1 Value of the Data

• The data provides insight into microbial diversity of biofilms on two distinct kinds of polymer namely polystyrene and nylon.
• The data will be useful for the scientific community working on the contamination of the aquatic environments due to microplastic and help them in understanding the diversity of bacteria in biofilms on the polymers and antibiotic resistance gene dissemination in the marine environment.

## 2 Data

**Fig. 1:**
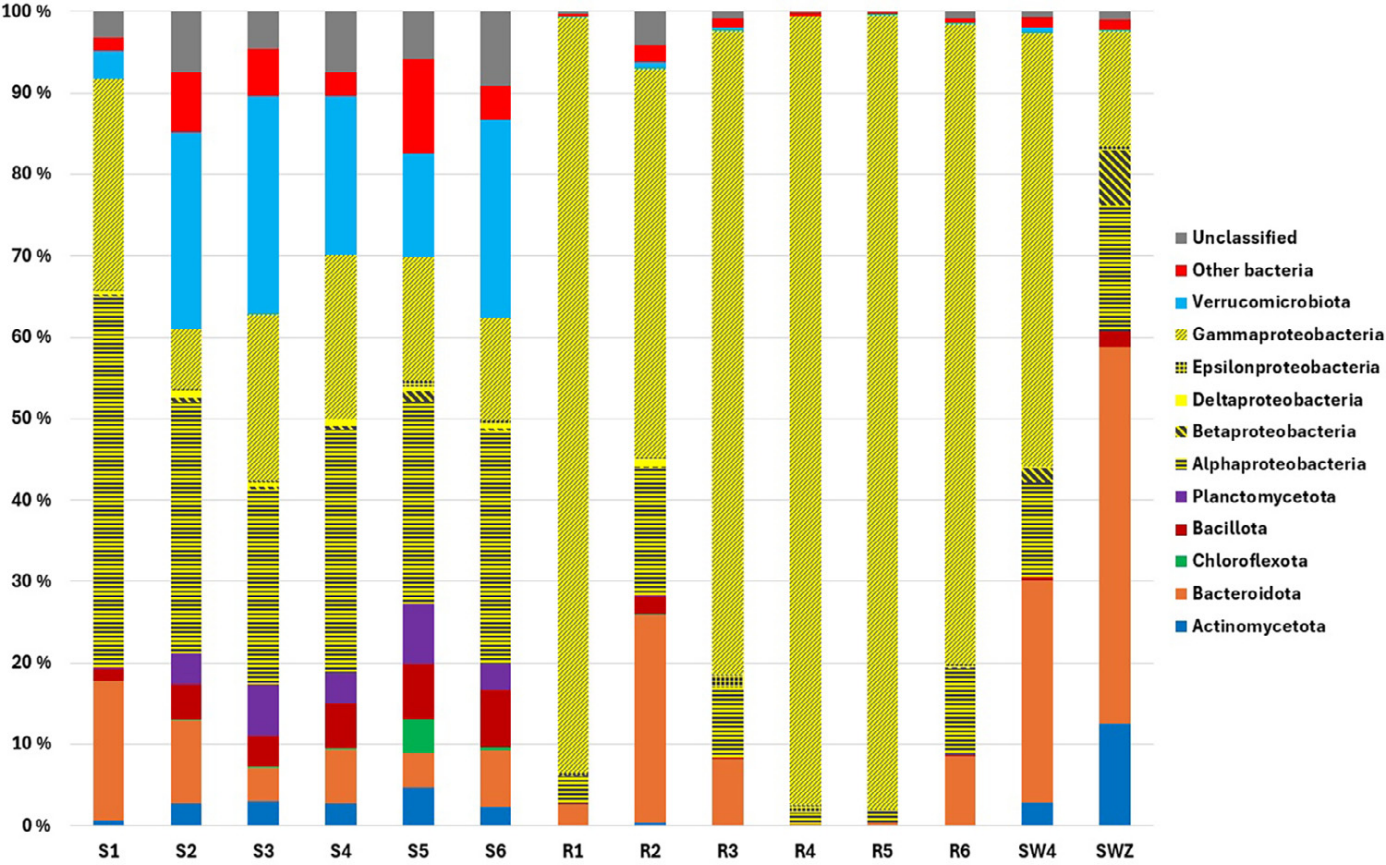
Bacterial composition of biofilms established on six polystyrene (S1–S6) and six nylon rope (R1–R6) surfaces. Seawater samples taken at day zero (SWZ) and after four weeks (SW4).

We obtained on average 124 million paired end reads per sample (ranging from 101-177 million reads per sample). In total, ∼460 Gigabases were analyzed from the shotgun metagenomic sequencing of the biofilm samples collected from polystyrene (S1-S6) and nylon ropes (R1-R6), and seawater taken at day zero (SWZ) and after four weeks (SW4). Based on 16S rRNA reads, a total of 20 phyla were detected in the biofilm samples collected from polystyrene samples, while 19 phyla were detected in the nylon rope samples (Fig. 1). At the phylum level, *Pseudomonadota* dominated in both polystyrene and nylon rope biofilms (average 49.3 % and 89.7 % relative abundance, respectively). The bacterial communities from nylon rope surfaces were dominated by the class *Gammaproteobacteria.* Most of the increase in *Gammaproteobacteria* could be assigned to the order of *Alteromonadales* and *Vibrionales*, which in turn received its major contributions from the families of *Pseudoalteromonadaceae* and *Vibrionaceae*, respectively. The bacterial communities from polystyrene surfaces showed a higher relative abundance of *Alphaproteobacteria.* Most of the increase in *Alphaproteobacteria* could be assigned to the order of Rhodobacterales, which in turn received its major contributions from the family of *Rhodobacteraceae*. The bacterial communities from SWZ were dominated by the class *Bacteroidota*, with major contribution from the family *Flavobacteriaceae*. The bacterial communities from SW4 were dominated by the class *Gammaproteobacteria*, with the family *Vibrionaceae* as the most dominant family. β-lactamaseswere the most abundant antibiotic resistance gene class (Supplementary Table S1). For nylon rope samples the second most abundant gene class detected were quinolone resistance genes. An average of 23 and 17 different β-lactamases were detected in polystyrene and the nylon rope biofilms, respectively.

Among the studied MGEs, integron-associated integrases (*intI)* were the most abundant detected from both polystyrene and nylon rope surfaces (average 0.0008 % and 0.011 % relative abundance, respectively). There was a higher abundance of *intI* genes in nylon rope samples, compared to polystyrene samples.

Several biocide resistance genes and metal resistance genes were detected from both polystyrene and nylon rope surfaces (Supplementary Table S1). Genes conferring resistance to a range of compounds, such as arsenic, cobalt, copper, lead, mercury, silver, and zinc, were found in higher abundance on nylon rope samples, compared to polystyrene samples. In contrast, genes conferring resistance to nickel were found in higher abundance in polystyrene samples.

Virulence factors (VFs) involved in adhesion factors, capsule formation, pilus formation, siderophore production, and toxin productions were also detected. Clinically relevant genes involved in toxin production (such as *cnf, toxA/B, bont, clbL/O/I*) and genes involved in siderophore production (such as *iucB, frgA, iroC*) were detected in the samples.

## 3 Background

Biofilms represent intricate assemblies of microorganisms that adhere to surfaces, forming a protective extracellular matrix. Plastic are prevalent materials in the environment, serve as optimal substrates for biofilm development. The formation of biofilms on synthetic polymers has attracted considerable attention due to their ecological and health implications. The process of biofilm formation on plastic surfaces typically unfolds in multiple stages. Initially, microorganisms attach to the surface through weak, reversible interactions. As they proliferate, they produce extracellular polymeric substances (EPS) that enhance their adhesion and establish a biofilm matrix [1,2]. This matrix offers protection against environmental stressors and antimicrobial agents. The composition of biofilms on plastic and polystyrene can vary based on environmental conditions. In aquatic environments, biofilms often comprise diverse bacterial species, algae, and fungi. Marine biofilms frequently include *Vibrio* spp., *Pseudomonas* spp, and various diatoms. In terrestrial settings, distinct microbial communities, including soil bacteria and fungi, may predominate [2]. Biofilms on plastic surfaces can exert both beneficial and detrimental effects. On one hand, they may contribute to the degradation of these materials, potentially facilitating the breakdown of plastic waste in the environment [3]. Certain microorganisms within these biofilms have demonstrated the capacity to utilize plastic as a carbon source, indicating a role in bioremediation. Conversely, biofilms can also serve as reservoirs for pathogenic microorganisms, potentially promoting the spread of diseases [1]. The presence of biofilms can modify the properties of plastic and polystyrene surfaces. They may increase surface roughness, affect hydrophobicity, and influence the material's susceptibility to degradation. In industrial contexts, biofilms on plastic surfaces can lead to biofouling, causing challenges in various applications, such as water treatment systems and food packaging [4,5]. Understanding the dynamics of biofilm formation on plastics is essential for developing strategies to mitigate their negative impacts and harness their potential benefits. This knowledge can inform the development of antifouling materials, enhance plastic waste management, and contribute to more effective bioremediation techniques.

## 4 Experimental Design, Materials, and Methods

**Fig. 2:**
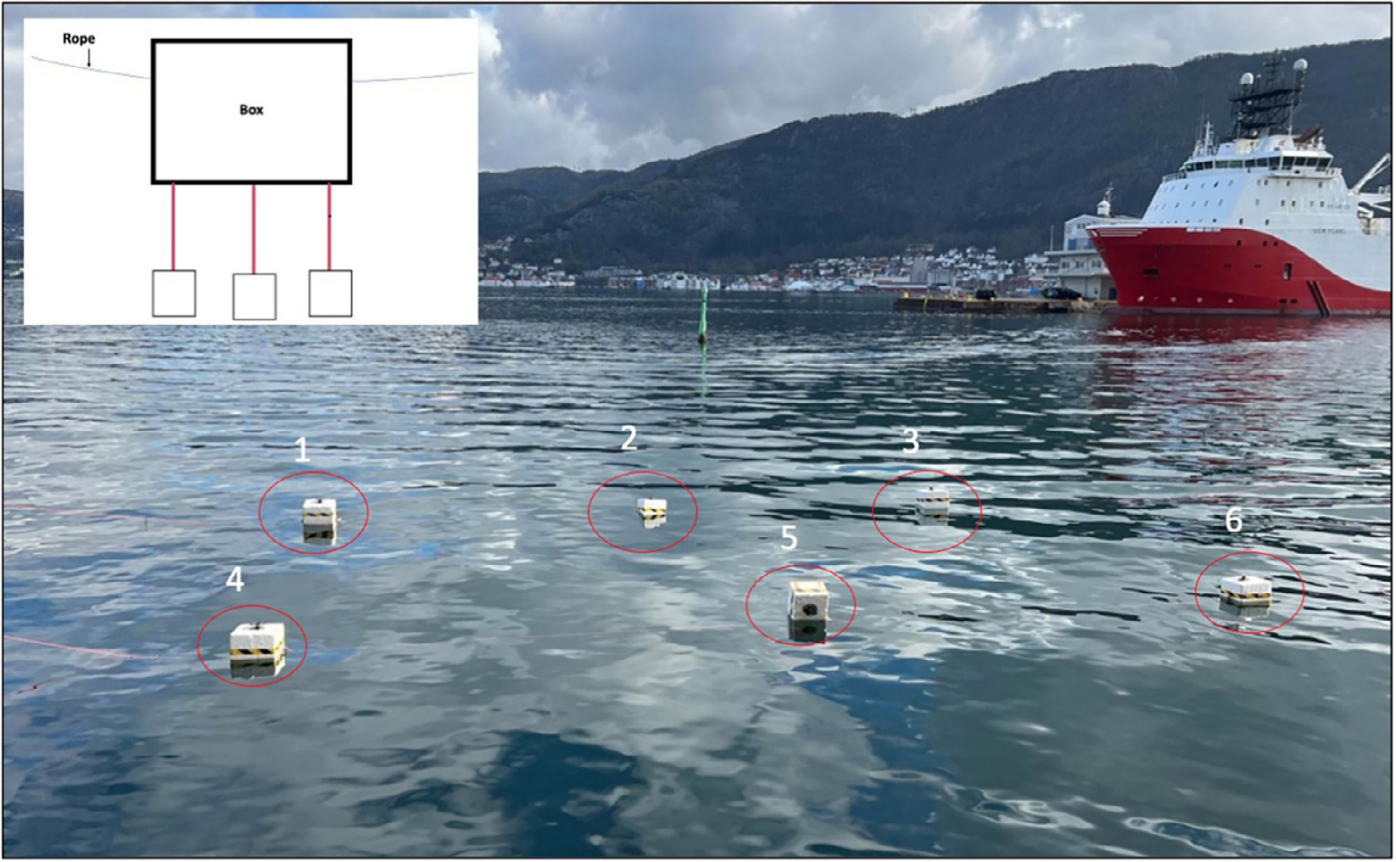
Overview of sampling set-up. Sampling site in Bergen, showing polystyrene boxes (numbered 1-6) where biofilms were collected. Left corner: Illustration demonstrating the attachment of ropes beneath the boxes.

The field experiment was conducted during Spring 2023 (April 4th 2023), outside the premises of the Institute of Marine Research in Bergen (60°40’04.35” N 5°30’63.33” E). During the sampling period, temperature and salinity measurements were performed weekly, using a conductivity-meter (VWR, Germany). Nylon ropes 4 mm (about 0.16 in) thickness and six polystyrene boxes were submerged in the sea with the help of metal plates for four weeks (Fig. 2). The boxes were placed at approximately 3 meters distance from each other. After four weeks (May 2nd, 2023), rope (n = 6) and polystyrene (n = 6) samples were collected and washed with sterile saline and the biofilms were collected, using a sterile plastic cell scraper (Thermo Fisher Scientific, USA), and stored in separate 15 mL sterile Falcon tubes (VWR, USA). The samples were stored at 4 °C and processed within 4 h of collection. The average seawater temperature during the experiment was 9 °C and the average salinity was 28.9 **‰** (Supplementary Table S2). In addition, 500 ml (about 16.91 oz) of seawater was collected on day 0 and 4 weeks later in sterile bottles and vacuum-filtered through S-Pak filters (pore size 0.22 µm) (Millipore, USA). Suspensions of biofilms were made in sterile phosphate buffer saline (PBS), 250 µL of the suspension was added to sterile tubes containing 180 µL of lysis buffer. Then the biofilm samples (n = 12) were incubated at 37 °C for 2 h. Subsequently, the samples were subjected to two rounds of bead beating, using a TissueLyzer (Qiagen, Germany). Two glass beads (4 mm diameter) were used for bead-milling at 20 Hz for 1 min. Following the bead beating step, the samples were subjected to another incubation at 37 °C for 40 min.

The biofilm on the rope was a bit darker in color than biofilm on polystyrene. Genomic DNA was extracted from the biofilm samples and seawater filters, using the DNeasy PowerWater kit (Qiagen, Germany) following the manufacturer’s protocol. The extracted DNA was quantified, using dsDNA High Sensitivity (HS) assay kit on the Qubit® Fluorometer (Thermo Fisher Scientific, USA). Shotgun metagenomic library were prepared, using Nextera Library Prep kit (Illumina, USA). Sequencing was performed, using Illumina NovaSeq platform (Illumina, USA), 150 bp paired end reads, at the Norwegian Sequencing Centre (Ullevål University Hospital, Oslo, Norway).

The sequences were quality-trimmed, using Trim Galore v.0.6.10 (http://www.bioinformatics.babraham.ac.uk/projects/trim_galore/) with a quality score of 30. The quality-processed reads from the metagenomes were mapped to a high-quality and manually curated database of antibiotic resistance genes [[6], [7], [8]], biocide resistance genes, metal resistance genes [9], mobile genetic elements [10], and virulence factors [11] using USEARCH usearch11.0.667_i86linux64[12]. For quantitative taxonomic distribution, quality-filtered shotgun reads were used as input to extract the reads corresponding to small subunit (SSU) 16S bacterial ribosomal RNA (rRNA) genes from the metagenomes and assigned them to different taxonomic groups, using Metaxa2 v.2.2.3 [13]. Relative abundance of each taxon from each taxonomic level (such as phyla and class) were calculated by calculating percentage of 16S rRNA gene sequence counts from each of the metagenomes. All metagenomes were down-sampled to 1,533,574,704 bp high quality-filtered reads for taxonomic assignment.

## CRediT Authorship Contribution

Conceptualization: NPM, Data curation: NPM, DHG, MPV Formal analysis: NPM, DHG, MPV Funding acquisition: NPM Investigation: NP, DHG, MP, TEL, VR, PSN Methodology: NPM, MP Project administration: NPM Resources: NPM Software: NPM, MP Supervision: NPM Validation: NPM Visualization: NPM, DHG, MPV Writing – original draft: NPM, DHG, MPV Writing – review & editing: NPM, DHG, MPV, TEL, VR, PSN

## Data Availability

NCBI SRAPlastic Styrofoam (Original data)

## Appendix Supplementary materials

Image, application 1

Image, application 2
