# Supplementary material for: High-throughput sequencing data of the microbiota and antibiotic resistance genes from biofilms on polystyrene and nylon rope incubated in Bergen harbor
Source: Data Brief. 2025 May 28;61:111718. doi: 10.1016/j.dib.2025.111718 (PMC12221519; doi:10.1016/j.dib.2025.111718)
Supplement: Supplementary file 2 [file mmc2.docx]

**Supplementary Table S1**. Measurements of temperature and salinity in seawater from the sampling site in Bergen, Norway 2024.

| **Date** | **Temperature (˚C)** | **Salinity (in ‰)** |  |  |
| --- | --- | --- | --- | --- |
| 04.04.24 | 8.8 | 26.1 |  |  |
| 09.04.24 | 8.2 | 27.0 |  |  |
| 13.04.24 | 8.4 | 33.1 |  |  |
| 18.04.24 | 10.7 | 29.3 |  |  |
